# Public awareness of cancer in Britain: a population-based survey of adults

**DOI:** 10.1038/sj.bjc.6605386

**Published:** 2009-12-03

**Authors:** K Robb, S Stubbings, A Ramirez, U Macleod, J Austoker, J Waller, S Hiom, J Wardle

**Affiliations:** 1 Cancer Research UK Health Behaviour Research Centre, Department of Epidemiology and Public Health, UCL, Gower Street, London WC1E 6BT, UK; 2 Cancer Research UK Promoting Early Presentation Group, Institute of Psychiatry, King's College London, St Thomas' Hospital, London, UK; 3 General Practice and Primary Care, Division of Community Based Sciences, Faculty of Medicine, 1 Horselethill Road, Glasgow, UK; 4 Cancer Research UK Primary Care Education Research Group, Cancer Epidemiology Unit, University of Oxford, Richard Doll Building, Roosevelt Drive, Oxford, UK; 5 Health Information Department, Cancer Research UK, 61 Lincoln's Inn Fields, London, UK

**Keywords:** cancer awareness, symptom awareness, anticipated delay, barriers, cancer warning signs

## Abstract

**Objective:**

To assess public awareness of cancer warning signs, anticipated delay and perceived barriers to seeking medical advice in the British population.

**Methods:**

We carried out a population-based survey using face-to-face, computer-assisted interviews to administer the cancer awareness measure (CAM), a newly developed, validated measure of cancer awareness. The sample included 2216 adults (970 males and 1246 females) recruited as part of the Office for National Statistics *Opinions Survey* using stratified probability sampling.

**Results:**

Awareness of cancer warning signs was low when open-ended (recall) questions were used and higher with closed (recognition) questions; but on either measure, awareness was lower in those who were male, younger, and from lower socio-economic status (SES) groups or ethnic minorities. The most commonly endorsed barriers to help seeking were difficulty making an appointment, worry about wasting the doctor's time and worry about what would be found. Emotional barriers were more prominent in lower SES groups and practical barriers (e.g. too busy) more prominent in higher SES groups. Anticipated delay was lower in ethnic minority and lower SES groups. In multivariate analysis, higher symptom awareness was associated with lower anticipated delay, and more barriers with greater anticipated delay.

**Conclusions:**

A combination of public education about symptoms and empowerment to seek medical advice, as well as support at primary care level, could enhance early presentation and improve cancer outcomes.

Patients with cancer in the United Kingdom tend to present with more advanced disease and have poorer survival rates than many of their European counterparts (Berrino *et al*, 2007; Sant *et al*, 2009). The most likely explanations for this are either late presentation by patients or late onward referral by general practitioners. Among patients with breast cancer, there is strong evidence from individual studies and systematic reviews of the world literature that delay between onset of symptoms and diagnosis/treatment is associated with poorer survival (Richards *et al*, 1999). Delay may result from patient, doctor and system factors (Andersen *et al*, 1995; Ramirez *et al*, 1999), and the Cancer Reform Strategy (Department of Health, 2007) has identified the need to investigate and target all of these factors to improve cancer outcomes.

The National Awareness and Early Diagnosis Initiative (NAEDI) consists of several work streams to help ensure delivery of the Cancer Reform Strategy. One of these has focused on developing a validated measure of public awareness of cancer signs and attitudes to help seeking, and benchmarking current levels on a national basis to provide a baseline against which to evaluate policy initiatives designed to improve awareness.

Two systematic literature reviews (Ramirez *et al*, 1999; MacDonald *et al*, 2004), investigating risk factors for patient delay in presenting with common cancers have shown the predominant risk factors to be lack of awareness of the seriousness of the symptom or not recognising that the symptom could be caused by cancer. If the symptom is atypical in nature, the risk of delayed presentation is increased.

The literature on cancer awareness goes back to the 1950s, and recent studies consistently indicate low public recognition of early warning signs (Brunswick *et al*, 2001; Grunfeld *et al*, 2002; McCaffery *et al*, 2003; Toon, 2007). However, most studies rely on *ad hoc*, non-validated measures. To the best of our knowledge, this study is the first to use a validated measure to assess awareness in a population-based sample. It examines disparities in relation to gender, age, socio-economic status (SES) and ethnicity, and investigates associations between awareness, perceived barriers and anticipated delay in presentation.

## Materials and methods

Data were collected as part of the Office for National Statistics *Opinions Survey* in September and October 2008. The *Opinions Survey* is considered a gold-standard system for recruiting a population-representative sample in Britain and is used for government data collection. Stratified probability sampling is used to select 67 postal sectors (sampling points) from the Postcode Address File of ‘small users,’ a database of ∼27 million private households in the United Kingdom receiving fewer than 50 items of mail per day. A random sample of addresses is chosen from each sampling point, which yielded a sample of 3652 households for the September and October surveys. For each household, the interviewer determines the household composition and identifies the respondent from among all adults aged over 16 using a Kish grid. The identified adult was invited to complete the cancer awareness measure (CAM) using a face-to-face, computer-assisted interview.

### Socio-demographic characteristics

The *Opinions Survey* includes a range of socio-demographic questions, of which the following are used in the present analyses: gender (male, female); age group (16–24, 25–34, 35–44, 45–54, 55–64, 65 and over); marital status (married/civil partnership, not married); ethnicity (white, other ethnic backgrounds); highest level of educational qualification obtained (degree or above, below degree, other, no formal qualifications); and occupation (managerial/professional, intermediate/small employers/lower supervisory, semi-routine/routine).

### Cancer awareness

The development process for the CAM is described elsewhere (Stubbings *et al*, 2009) but briefly, items were developed using the existing literature, a search of unpublished reports, and input from an expert advisory panel. These were then modified iteratively through expert consensus, following which item analysis was used to reduce the item pool. Interviews with the general public in which respondents were encouraged to verbalise their cognitions as they responded to each item were used to establish that the questions were interpreted as intended. Test–retest reliability was assessed with repeat administration over a 2-week interval (mean *r*=0.81). External validity was established by demonstrating that a group of cancer experts (not involved in the CAM development) scored significantly higher than a group of equally educated non-experts (historians and linguists). Sensitivity to change in knowledge was demonstrated by showing that scores obtained by members of the general public were significantly higher after a brief educational intervention.

Data reported here are on awareness of warning signs, anticipated time before seeking medical help and perceived barriers to presentation for nine common warning signs. Results are presented in the order in which questions were asked during the interview.

#### Awareness of cancer warning signs

Awareness of cancer warning signs was assessed with both ‘open’ and ‘closed’ questions; neither is perfect but the sources of bias are different. Open questions estimate the extent to which cancer signs can be brought to mind, and reflect what is more usually thought of as knowledge, but performance also depends on memory and perseverance in the recall task. Closed questions test recognition of symptoms and avoid recall problems, but are potentially biased by the respondents' expectation about whether the signs listed are likely to be valid, and encourage guessing. Some closed measures include ‘distractor items’ but we chose not to include such items in the CAM because of the difficulty in identifying signs that are definitely not associated with cancer, and uncertainty over whether distractor endorsement should be counted negatively against the final score (given that cancers can manifest in many ways and a respondent could have experience of a cancer presenting with a symptom we had designated a distractor). Our previous work has shown that closed questions produce a higher awareness score than open questions, but the correlates of the two types of question tend to be similar (Waller *et al*, 2004).

The open-ended awareness item was phrased as: ‘There are many warning signs and symptoms of cancer. Please name as many as you can think of’ (For discussion about the decisions on wording of questions, see the CAM development paper (Stubbings *et al*, 2009)). Interviewees were prompted with ‘anything else’ until no further answers were provided. The closed question said: ‘The following may or may not be warning signs for cancer. We are interested in *your* opinion.’ This was followed by a list of the nine warning signs from Cancer Research UK's leaflet *Cancer – know the warning signs* (http://publications.cancerresearchuk.org/WebRoot/ crukstoredb/CRUK_PDFs/RTR200.pdf). We combined items on changes in bowel or bladder habits to reduce participant burden. Cancer Research UK has since change their list to include 12 signs. The nine signs listed in the CAM were: lump or swelling, persistent unexplained pain, unexplained bleeding, persistent cough or hoarseness, persistent change in bowel or bladder habits, difficulty swallowing, change in the appearance of a mole, a sore that does not heal and unexplained weight loss. The open-ended question was always asked before the closed questions to reduce bias. For both types of question, the number of warning signs endorsed was summed to produce total scores.

#### Anticipated delay

Closed questions were used to assess anticipated help seeking for each of the symptoms (‘If you had […], how soon would you contact your doctor to make an appointment to discuss it?’). Response options ranged from ‘1 to 3 days’ to ‘Never.’ For some analyses, response categories were combined into two categories of lower anticipated delay (<2 weeks) *vs* higher anticipated delay (2 weeks or more) (We recognise that <2 weeks is fast, but decided that it represented a reasonable dividing line between prompt action and a degree of procrastination.). Anticipated delay was highly correlated across symptoms and principal components analysis showed that anticipated delay for all nine symptoms loaded on one main factor. We therefore calculated the total number of symptoms for which anticipated delay was under 2 weeks, and this score was used as the outcome for some analyses.

#### Barriers to help seeking

Barriers to help seeking were assessed with 10 items identified in the general primary care literature. They included four emotional barriers (e.g. worried what the doctor might find), three practical barriers (e.g. too busy) and three service barriers (e.g. not wanting to waste the doctor's time). Response options were ‘Yes often,’ ‘Yes sometimes’ and ‘No,’ which for some analyses were re-categorised as ‘yes’ or ‘no.’ Summation of ‘yes’ responses was used to identify a total number of barriers.

### Analysis

Data were analysed using SPSS 14.0. Descriptive statistics were completed for gender, age, marital status, ethnicity and occupational category (SES) and items from the CAM. *χ*^2^ tests and analysis of variances were used to examine relationships between demographic characteristics and CAM items. Analysis of covariance (ANCOVA) was used to examine the relationship between demographic factors and awareness of cancer warning signs assessed by recall (open) and recognition (closed) questions. ANCOVA was also used to examine independent predictors of anticipated delay. There were very few missing items on the CAM (average 12 cases for any question). One hundred and eighty-one were unclassified as to occupation and were excluded from the multivariate analyses that included SES.

## Results

Of 3652 households invited to participate, 2216 (61%) respondents agreed to be interviewed, 1093 (30%) refused and 324 (8%) could not be contacted after three attempts. Of the 2216 people who took part in an interview, eight (0.4%) did not answer any questions from the CAM and so are excluded from the sample. Respondent demographics approximated the British population but with a trend towards higher levels of education and occupational status (see Table 1).

### Recall and recognition of cancer warning signs

**Figure 1:**
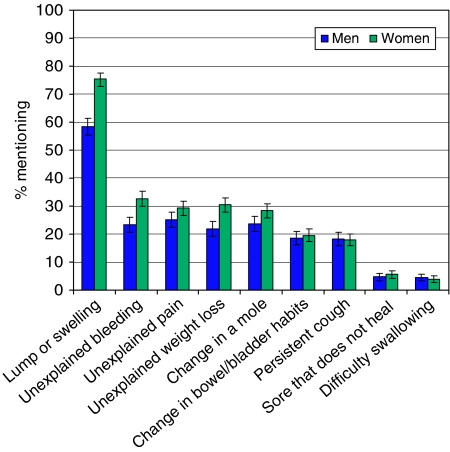
Recall of nine warning signs.

Recall (open question) was good for the classic tumour symptom of lump/swelling (68%), but very poor for all other symptoms (e.g. 5% for a sore that does not heal). Figure 1 shows recall for each warning sign by gender. Overall, men recalled 2.0 (±1.7) signs and women recalled 2.4 (±1.6) (t(2194)=6.43, *P*<0.001).

Recognition (closed items) gave a considerably higher score than recall. Change in the appearance of a mole and lump/swelling were the most recognised (both 94%), and even the least recognised sign (a sore that does not heal) was acknowledged by over 60% of participants. However, there was still an SES gradient for each warning sign, with the highest SES group recognising a total of 7.6 (±1.9) signs compared with 6.9 (±2.2) in the lowest SES group (F(2,2015)=20.31, *P*<0.001). Women recognised 7.4 (±2.0) signs compared with men's 7.0 (±2.2) (t(2195)=4.99 *P*<0.001). White participants recognised 7.3 (±2.0) warning signs, while respondents from other ethnic backgrounds recognised 6.2 (±2.9) (t(2195)=6.22, *P*<0.001). In relation to age, respondents aged 55–64 years reported the most (7.8±1.7), and those aged 16–24 reported the fewest (6.1±2.1; F(5,2196)=22.12, *P*<0.001).

Table 2 shows multivariate analyses for the recall and recognition of the nine cancer warning signs. In an ANCOVA assessing number of warning signs recalled, women recalled significantly more than men, older people did better than younger people, and married people recalled more signs than those who were not married. There was a strong SES gradient, with higher SES groups recalling significantly more symptoms. Ethnic minorities had lower symptom recall than white respondents; an association that persisted after controlling for SES.

In an ANCOVA of the total number of cancer warning signs recognised, being female, older, married, white, and in a higher SES group, were significant independent predictors (see Table 2).

### Barriers to help seeking

The most widely endorsed barriers to consultation were difficulty making an appointment (37% men, 45% women), not wanting to ‘waste the doctor's time’ (36% men, 41% women) and worry about what the doctor might find (34% men, 40% women), but all items were endorsed to some extent (see Table 3). Grouping the barriers into emotional, practical and service indicated that lower SES respondents endorsed more emotional barriers – being worried about what the doctor might find, embarrassed and not confident in talking to the doctor about the symptom. Higher SES respondents were more likely to report practical barriers (too busy, having other things to worry about). There were no SES differences in service barriers. All barriers were equally endorsed by white and ethnic minority groups with the exception of not wanting to ‘waste the doctor's time’ where 40% of white respondents endorsed this item compared with only 24% of ethnic minorities (*χ*^2^(1,2174)=13.16, *P*<0.001).

### Anticipated delay

The majority of respondents indicated that they would seek medical help in <2 weeks for most symptoms (see Table 4). Lower SES respondents reported less anticipated delay than higher SES respondents for each of the warning signs.

The relationship between anticipated delay and age was examined by looking at the total number of symptoms for which respondents would wait 2 weeks or more before seeking help. The youngest age group and the oldest group reported the lowest anticipated delay (16–24 years: 3.90±2.71 and 65+ years: 3.77±2.67), with the age groups in between reporting greater anticipated delay (25–34: 4.46±2.64, 35–44: 4.48±2.73, 45–54: 4.33±2.78, 55–64: 4.01±2.69; F(5,2207)=5.22, *P*<0.001).

### Associations between awareness, perceived barriers and anticipated delay

In an ANCOVA, including the number of warning signs identified and the number of barriers endorsed, perceiving more barriers to help seeking was associated with greater anticipated delay (F(1,2008)=91.70, *P*<0.001). Recall (open question) was not associated with anticipated delay, but recognising more symptoms was associated with lower anticipated delay independently of gender, age, ethnicity, occupation and perceived barriers (F(1,2008)=4.93, *P*<0.02). Significant independent effects were maintained for gender (women: adjusted mean=3.58±0.13, men: 3.97±0.14; F(1,2008)=11.41, *P*=0.001). Being from an ethnic minority group (ethnic minority: 3.42±0.24, white: 4.14±0.06; F(1,2008)=8.58, *P*=0.003) or a lower SES background (lowest SES group: 3.14±0.15, highest SES group: 4.32±0.14; F(2,2008)=36.36, *P*<0.001) was associated with less anticipated delay.

## Discussion

In reviewing the literature we found no other study using a validated measure to assess cancer awareness in a population-based sample. In this British, population-based sample, recall of cancer warning signs using an open question was relatively poor (<30%) for all symptoms except ‘lump/swelling,’ which was mentioned by 68% of respondents. Recognition of cancer warning signs with a closed question was much higher, with ‘mole’ and ‘lump/swelling’ being identified by over 90% of participants. The higher levels of recognition for those two warning signs may reflect the success of breast and skin cancer awareness-raising campaigns (e.g. Breast Cancer Awareness Month and the SunSmart Campaign – http://www.sunsmart.org.uk).

We predicted that recognition scores would be greater than recall scores (Waller *et al*, 2004), but it is difficult to determine which better captures the concept of cancer awareness. Recall underestimates awareness because it is limited by memory, while recognition overestimates awareness because participants find it easy to guess. However, recall and recognition had similar correlates, both being higher in respondents who were female, older, white and from higher SES backgrounds. Ajzen and Fishbein (2000) argue that what is important in predicting attitudes, intentions and behaviour is the salience or accessibility of beliefs, the most accessible beliefs being those that can be readily brought to mind: ‘*people's attitudes follow spontaneously and consistently from beliefs accessible in memory and then guide corresponding behavior.*’ Applying their proposal to our data would suggest that symptoms that are recalled in response to open-ended questions are more likely to lead to help seeking than those that are merely recognised. However, there is a need for further investigation of the ways in which different approaches to measuring cancer knowledge relate to behavioural outcomes, and to determine the most useful measures for predicting early detection behaviours.

Most respondents anticipated little delay in seeking medical help if they noticed a cancer warning sign, saying that they would contact their doctor within 2 weeks for the majority of symptoms. Lower SES and ethnic minority groups reported *less* anticipated delay, a finding inconsistent with systematic reviews showing lower levels of education and non-white ethnicity to be associated with longer delay (Ramirez *et al*, 1999; Mitchell *et al*, 2008) but consistent with the observation that some reported barriers to help seeking were lower in these groups. While these results are encouraging both in terms of general help-seeking behaviour and inequalities, they are severely limited by their hypothetical nature. The gap between good intentions and behaviour is well recognised in the psychological literature (Sheeran, 2002), and actual help seeking is likely to be less prompt than hypothetical intentions.

Being worried about what the doctor might find was the most commonly endorsed emotional barrier to prompt help seeking, which is in line with previous work citing fear and fatalism as barriers to cancer-protective behaviours (Powe, 1995; Aro *et al*, 2001; Lostao *et al*, 2001; Subramanian *et al*, 2004). But it was also notable that almost 40% of people felt that concern about ‘wasting the doctor's time’ could make them delay presentation. This suggests that some people may not feel confident that their symptom needs medical attention or perceive their doctor as too busy to be bothered with their concerns. Either way, it should be possible to address this issue through primary care initiatives that empower people to believe their symptom is important and deserving of medical attention. The most endorsed barrier of all was ‘difficult to make an appointment,’ and this perception should change as primary care services continue to improve.

Recognising more warning signs was related to lower anticipated delay independently of SES, ethnicity, age, gender and perceived barriers. This is consistent with the idea that awareness of cancer warning signs could ultimately contribute to earlier detection of cancer. In contrast, recall of cancer warning signs was not associated with anticipated delay, despite having many of the same demographic correlates. This has some ecological validity in that it may be less important for people to be able to recall the nine warning signs than to recognise a symptom as serious once they notice it. Equally, it could relate to the question formats: both recognition and delay questions were presented as a series of nine symptoms, which could in part explain why recognition showed closer associations with delay than did recall.

Age showed significant associations with both recall and recognition of warning signs, such that scores increased with increasing age up to 64 years. However, the oldest age group (65 years and over) had lower recall and recognition, which is interesting and concerning, given that this group is at highest risk of cancer. This finding may reflect memory loss or cognitive impairments in this group (mean age was 75 years with a range of 65–101), or could reflect their greater lifetime experience of possible cancer symptoms, which have proved benign. An alternative explanation might be that they have never been made aware of the warning signs because cancer would have been discussed less when they were younger. Further work is needed to explore this in greater detail.

This study has strengths and weaknesses. One strength is the use of a population-based sample. Although the response rate was only 61% and we do not know how the remaining 39% would score on cancer awareness, it is in line with other population-based contemporary surveys. In addition, some cases (∼8%) were excluded from analyses because they could not be classified for SES, which could bias the results. Fortunately, there were few missing data on the CAM questions, and therefore responses are representative of the survey respondents, but generalisation beyond British adults cannot be assumed.

A second strength is the use of a validated measure of cancer awareness, but nonetheless there is no perfect measure, and both the recall and recognition questions have limitations, as discussed. Relying on hypothetical questions to assess delay revealed surprisingly prompt help-seeking intentions, which is likely to be an overestimate compared to real-life situations with all their uncertainties and competing priorities. However, this indicates that people are intuitively aware of the importance of prompt presentation, and therefore that interventions to facilitate this should fall on fertile ground. The order of the questions in the CAM may have an impact on the findings, particularly the fact that cancer symptoms are listed in the recognition questions before asking about anticipated delay – this may have the effect of priming respondents to say that they would present promptly. However, in most situations it is not pragmatically feasible to randomise the order in which the questions are asked, and possible priming effects were considered when designing the measure.

A weakness of the study was that because cancer is so strongly related to increasing age, many respondents were at relatively low risk due to their young age. Thus, the results may not be fully applicable to the older, most at-risk group.

If the CAM is used nationally and internationally, it will provide an exciting opportunity for researchers to compare levels of awareness between countries and over time. Use of the CAM should aid health educators in identifying subgroups within the population with lower levels of cancer awareness. In addition, evaluation of cancer awareness-raising campaigns will benefit from a validated measure. Further work is needed to explore the reasons for patient delay in presenting with cancer symptoms because measuring awareness is only the first step in beginning to understand this process. Work is under way to develop an additional module for the CAM, which will measure beliefs and attitudes about cancer and provide insights into predictors of cancer preventive behaviours.

Overall, it seems that whether cancer awareness is assessed by recall or recognition there is room for improvement in levels of public awareness particularly among men, lower SES groups and those from ethnic minorities. If the objectives of NAEDI are to be achieved, the public needs not only to be able to recall and recognise warning signs but also to understand their potentially serious significance and avoid delay in seeking medical help. A combination of public education about symptoms, empowering people to seek medical advice and providing positive information about the value of early detection could enhance early presentation and improve cancer outcomes.

## Conflict of interest

The authors declare no conflict of interest.

## Figures and Tables

**Table 1:** Demographic characteristics of sample (*n*=2208)

	***N***	**%**
*Gender*		
Male	968	43.8
Female	1240	56.2
		
*Age (years)*		
16–24	170	7.7
25–34	323	14.6
35–44	382	17.3
45–54	310	14.0
55–64	397	18.0
65 and over	626	28.4
		
*Marital status*		
Married/civil partnership	984	44.6
Not married	1224	55.4
		
*Ethnicity*		
White	2064	93.5
Other ethnic backgrounds	144	6.5
		
*Occupation (SES)*		
Managerial/professional (higher SES)	744	33.7
Intermediate/small employers/lower supervisory (mid SES)	626	28.4
Semi-routine/routine (lower SES)	657	29.8
Not classified	181	8.2
		
*Highest qualification obtained*		
Degree or above	368	16.7
Below degree	791	35.8
Other	254	11.5
No formal qualifications	343	15.5
Missing data	452	20.5

Abbreviation: SES=socio-economic status.

**Table 2:** Analysis of covariance for recall and recognition of the nine cancer warning signs

	**Recall (open question)**		**Recognition (closed question)**	
**Demographic groups**	**Mean (95% CI)**	***P***	**Mean (95% CI)**	***P***
*Gender*				
Male	1.64 (1.47, 1.80)		6.47 (6.25, 6.68)	
Female	2.20 (2.04, 2.36)	F(1,2014)=64.10, *P*<0.001	7.02 (6.82, 7.23)	F(1,2015)=38.41, *P*<0.001
				
*Age (years)*				
16–24	1.49 (1.16, 1.83)		5.90 (5.46, 6.33)	
25–34	1.72 (1.51, 1.93)		6.48 (6.21, 6.75)	
35–44	1.90 (1.69, 2.10)		6.63 (6.37, 6.89)	
45–54	2.08 (1.87, 2.30)		7.10 (6.82, 7.37)	
55–64	2.51 (2.30, 2.72)		7.39 (7.12, 7.66)	
65 and over	1.80 (1.62, 1.99)	F(5,2014)=13.38, *P*<0.001	6.97 (6.73, 7.21)	F(5,2015)=13.15, *P*<0.001
				
*Marital status*				
Married	2.07 (1.90, 2.24)		6.88 (6.67, 7.10)	
Not married	1.77 (1.60, 1.93)	F(1,2014)=17.49, *P*<0.001	6.60 (6.39, 6.81)	F(1,2015)=9.28, *P*=0.002
				
*Ethnicity*				
White	2.21 (2.12, 2.29)		7.16 (7.06, 7.27)	
Other ethnic backgrounds	1.63 (1.34, 1.91)	F(1,2014)=14.95, *P*<0.001	6.32 (5.96, 6.69)	F(1,2015)=19.26, *P*<0.001
				
*Occupation (SES)*				
Managerial/professional (higher SES)	2.31 (2.14, 2.49)		7.13 (6.90, 7.35)	
Intermediate/small employers/lower supervisory (mid SES)	1.86 (1.68, 2.05)		6.70 (6.47, 6.94)	
Semi-routine/routine (lower SES)	1.58 (1.40, 1.76)	F(2,2014)=38.45, *P*<0.001	6.40 (6.18, 6.63)	F(2,2015)=22.43, *P*<0.001

Abbreviation: SES=socio-economic status.

**Table 3:** Emotional, practical and service barriers to seeking medical help (% endorsing each) by socio-economic group (indexed by occupational category)

	**All (*n*=2208)**	**Lower SES (*n*=662)**	**Mid SES (*n*=627)**	**Higher SES (*n*=746)**	**Significance**
*Emotional barriers*					
Worried what doctor might find	36.5 (807)	44.1 (283)	35.2 (217)	33.2 (243)	*χ*^2^(1, 1989)=17.08, *P*<0.001
Too scared	24.8 (547)	26.4 (168)	25.7 (158)	23.3 (169)	*χ*^2^(1, 1977)=1.82, *P*=0.177
Too embarrassed	20.5 (452)	25.5 (164)	19.4 (119)	15.6 (115)	*χ*^2^(1, 1993)=20.74, *P*<0.001
Not confident to talk about symptom	11.8 (260)	13.9 (89)	10.7 (66)	10.1 (74)	*χ*^2^(1, 1992)=4.77, *P*=0.029
					
*Practical barriers*					
Too busy	28.4 (626)	19.6 (127)	26.9 (167)	38.3 (282)	*χ*^2^(1, 2005)=59.0, *P*<0.001
Other things to worry about	21.7 (480)	17.6 (113)	21.7 (134)	26.4 (194)	*χ*^2^(1, 1996)=15.34, *P*<0.001
Difficult to arrange transport	4.7 (103)	6.6 (43)	4.8 (30)	2.8 (21)	*χ*^2^(1, 2010)=11.13, *P*=0.001
					
*Service barriers*					
Difficult to make appointment	40.7 (899)	41.6 (266)	40.7 (251)	43.3 (315)	*χ*^2^(1, 1983)=.41, *P*=0.522
Worried about wasting doctor's time	38.1 (842)	39.4 (251)	42.7 (265)	36.4 (269)	*χ*^2^(1, 1995)=1.44, *P*=0.229
Difficult to talk to doctor	13.4 (296)	14.5 (90)	14.2 (86)	12.5 (89)	*χ*^2^(1, 1938)=1.15, *P*=0.283

Abbreviation: SES=socio-economic status.

**Table 4:** Percentage saying that they would contact the doctor in <2 weeks for each warning sign by socio-economic group (indexed by occupational category)

	**Lower SES (*n*=662)**	**Mid SES (*n*=627)**	**Higher SES (*n*=746)**	**Significance**
*Warning signs % (n)*				
Unexplained bleeding	95.3 (614)	91.9 (564)	92.0 (674)	*χ*^2^(1, 1991)=5.82, *P*=0.016
Difficulty swallowing	85.6 (545)	79.2 (488)	73.8 (542)	*χ*^2^(1, 1987)=28.41, *P*<0.001
Lump or swelling	83.4 (534)	76.6 (472)	73.0 (534)	*χ*^2^(1, 1988)=21.26, *P*<0.001
Change in appearance of a mole	82.8 (519)	74.2 (451)	71.2 (521)	*χ*^2^(1, 1967)=24.24, *P*<0.001
Unexplained pain	78.5 (499)	71.5 (434)	67.5 (487)	*χ*^2^(1, 1965)=20.24, *P*<0.001
Sore that does not heal	70.2 (447)	57.8 (354)	54.1 (394)	*χ*^2^(1, 1977)=35.84, *P*<0.001
Change in bowel/bladder habits	70.7 (451)	59.2 (362)	50.6 (371)	*χ*^2^(1, 1982)=56.87, *P*<0.001
Cough or hoarseness	56.3 (359)	45.4 (278)	37.5 (275)	*χ*^2^(1, 1984)=48.32, *P*<0.001
Unexplained weight loss	50.8 (318)	34.1 (207)	27.4 (200)	*χ*^2^(1, 1963)=77.73, *P*<0.001

Abbreviation: SES=socio-economic status.
